# Lessons learned from the first European project on the integration of infectious diseases in testing services, data collection and country responses

**DOI:** 10.1186/s12879-021-06362-7

**Published:** 2021-09-13

**Authors:** Dorthe Raben, Jordi Casabona, Lella Cosmaro, Nadia Gasbarrini, John S. Lambert, Shannon Glapsy, Christine Kakalou, Manuel Maffeo, Michele Mommi, Gianmarco Corradini, Irena Klavs, Aljona Kurbatova, Iwona Wawer, Piotr Wysocki, Anne Raahauge, Stine Finne Jakobsen, Jeffrey V. Lazarus, Ann Sullivan, Meaghan Kall, Dagmar Hedrich, Cheryl Case Johnson, Nicole Simone Seguy, Daniel Simões, Valerie Delpech

**Affiliations:** 1 CHIP, Centre of Excellence for Health, Immunity and Infections (CHIP), Rigshospitalet, University of Copenhagen, Blegdamsvej 9, DK-2100 Copenhagen, Denmark; 2 Centre for Epidemiological Studies on STI/HIV/AIDS in Catalonia (CEEISCAT), Institut Josep Carreras, Campus de Can Ruti, Ctra de Can Ruti, Camí de les Escoles, s/n, 08916 Badalona, Catalonia Spain; 3 CIBER Epidemiologia y Salud Pública (CIBERESP), Madrid, Spain; 4 Fondazione LILA Milano - Italian League for Fighting AIDS, Via Carlo Maderno 4, 20136 Milan, MI Italy; 5 Fondazione Villa Maraini, Via Bernardino Ramazzini, 31 -, 00151 Rome, RM Italy; 6 Mater Misericordiae University Hospital and UCD School of Medicine, Belfield, Dublin 4, Ireland; 7 Centre for Research & Technology Hellas, Institute of Applied Biosciences, 6th km Charilaou-Thermi Rd, P.O. Box 60361, 57001 Thessaloniki, GR Greece; 8 Arcigay Associazione LGBTI Italiana, Via Don Minzoni 18, 40121 Bologna, Italy; 9 National Institute of Public Health, Trubarjeva 2, 1000 Ljubljana, Slovenia; 10 National Institute for Health Development, Hiiu 42, 11619 Tallinn, Estonia; 11 National AIDS Centre, Agency of the Ministry of Health, Samsonowska 1, 02-829 Warszawa, Poland; 12 Barcelona Institute for Global Health (ISGlobal), Hospital Clínic, University of Barcelona, Calle del Rossellon 132, ES-08036 Barcelona, Spain; 13 Chelsea and Westminster Hospital NHS Foundation Trust, 369 Fulham Road, London, London SW10 UK; 14 Public Health England, Wellington House 133-155 Waterloo Road, London, SE1 8UG UK; 15 European Monitoring Centre for Drugs and Drug Addiction (EMCDDA), Praça Europa 1, Cais do Sodré, 1249-289 Lisbon, Portugal; 16 World Health Organisation (WHO) Regional Office for Europe, FN Byen, Marmorvej 51, 2100 Copenhagen, Denmark; 17 World Health Organisation (WHO) HQ, Avenue Appia 20, 1202 Geneva, Switzerland; 18 Grupo de Ativistas em Tratamentos (GAT), Avenida Paris, 4 – 1 Direito, 1000-228 Lisbon, Portugal

**Keywords:** HIV, Viral hepatitis, Sexually transmitted infections, Tuberculosis, Integrated services, Europe

Despite the progress in effective treatments for HIV, viral hepatitis, tuberculosis and sexually transmitted infections (STIs), these infections remain major public health concerns across Europe. Recurring challenges of late presentation and unprioritized prevention programmes need to be effectively addressed in order to control and prevent transmission and ensure that people are diagnosed early and rapidly enter the care system. The prevalence of co-infections is high due to the social context of key populations and the shared modes of transmission, varying with local epidemiology, which underlines the need to combine efforts throughout the continuum of care.

In its third Health Programme (2014–2020) the European Commission endorsed and prioritized a cross-disease integrated approach to combine efforts and promote cost-effective, affordable and effective interventions. Infectious diseases are generally managed in parallel structures with disease specific policies and actions (e.g. the 2014–2016 Action Plan on HIV/AIDS) at both European and national levels [1, 2]. In this framework, a three-year Joint Action on integrating prevention, testing and linkage to care strategies across HIV, viral hepatitis, TB and STIs in Europe (INTEGRATE) was launched in Europe in 2017 [3, 4]. National Ministries of health in 16 European Union (EU) and neighbouring countries nominated 29 organisations (from non-governmental institutions (NGOs) to public health institutes and hospitals), to form a consortium focused on the integration of early diagnosis and linkage to prevention and care of HIV, viral hepatitis, TB and STIs.

The approach in INTEGRATE has been to explore how effective tools for diagnosis and linkage to prevention and care for one disease can be used for other diseases; the applied methodology was to review existing tools and then adapt and pilot these tools in other disease areas (Fig. 1). A baseline survey was conducted in late 2017 to map partner and country level testing activities, monitoring and surveillance data collection, treatment and linkage to care, prevention activities and training needs.

**Fig. 1:**
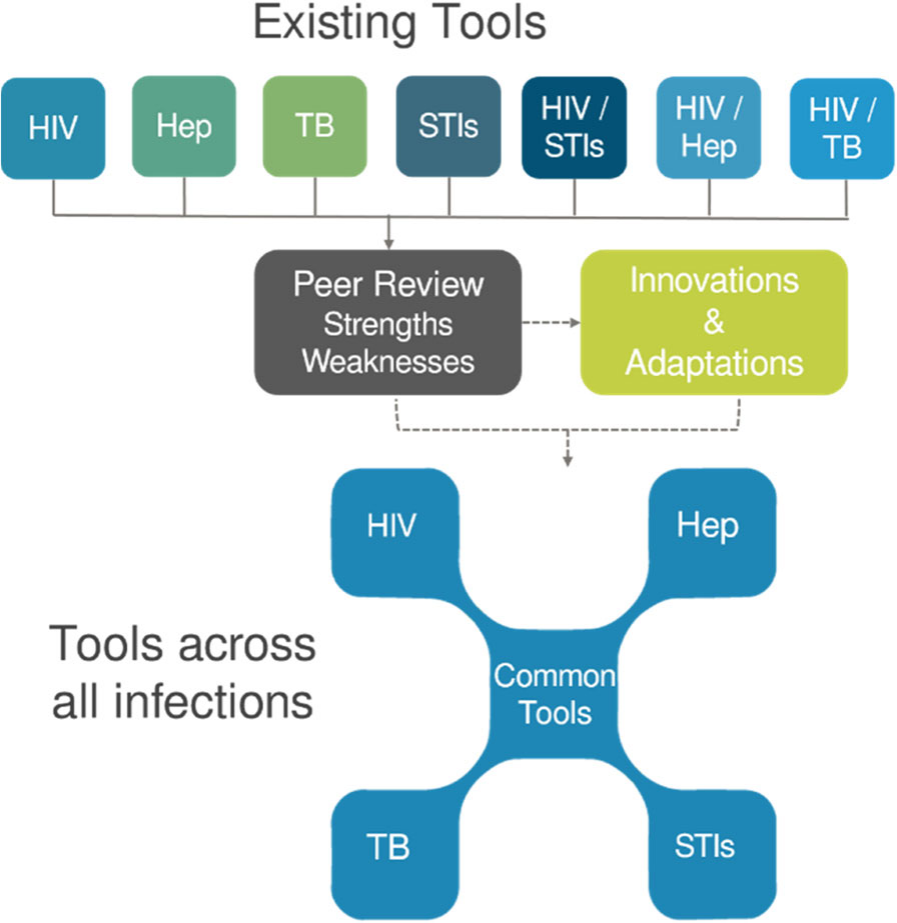
INTEGRATE review methodology. Existing effective tools for diagnosis and linkage to prevention and care were reviewed, adapted and piloted in other disease areas

The project built upon the experience and knowledge of other European testing-oriented projects from previous Health Programs such as HIV-COBATEST, Euro HIV EDAT and OptTEST, which had focussed primarily on optimising testing for HIV, as well as the EuroTEST initiative and its related projects [5–7].

INTEGRATE has been organised with 4 horizontal and 4 core and complementary work packages (Fig. 2), reflected in the different research articles presented in this supplement. This article summarises the overall main outcomes of the INTEGRATE project.

**Fig. 2:**
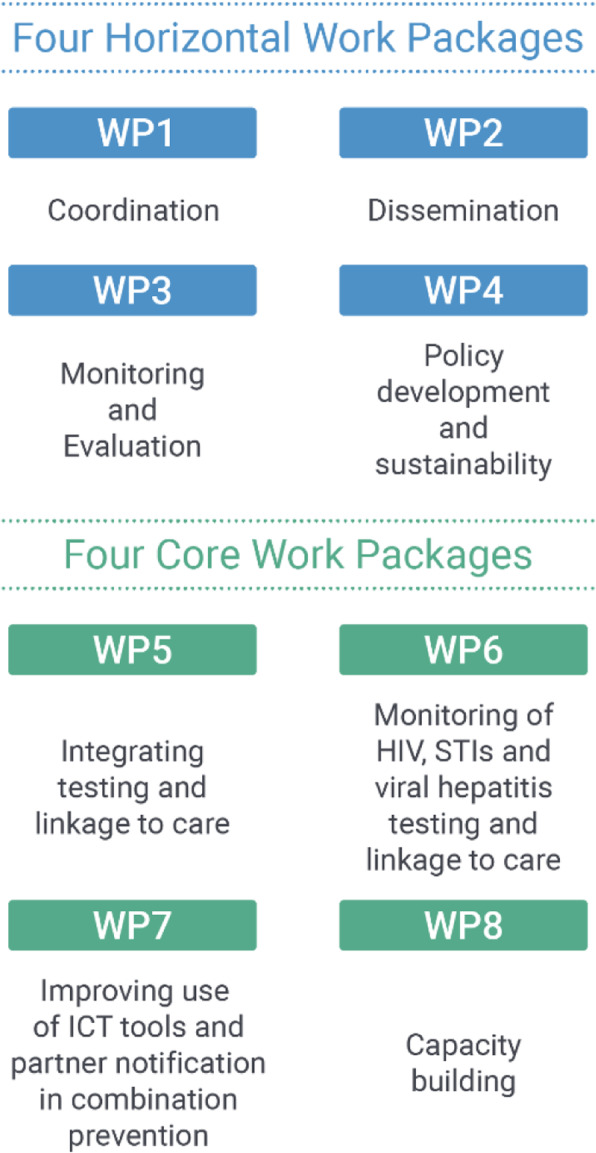
INTEGRATE Work Packages

## Integrated testing

An important focus area of INTEGRATE has been to investigate missed opportunities for combined testing for HIV, viral hepatitis, STIs and - where relevant – TB. Studies and testing services designed for HIV testing have demonstrated the benefits of introducing a combination of tests for these infections depending on target group and service set-up. Advances in HIV testing approaches have been explored to examine how they could potentially be expanded to include testing for other diseases and to evaluate their effectiveness. The European Testing Week (ETW) [8] was expanded to also advocate for raising testing awareness for viral hepatitis and STIs and INTEGRATE successfully launched a pilot Spring ETW that included viral hepatitis in May 2018 to complement the original November campaign running since 2013 the week before World Aids Day (1 December). The pilot observed an increase in the proportion of involved organizations implementing combined activities (targeting more than one disease) from 50% during the previous November ETW in 2017 to 64% during the pilot. Furthermore, the concept of testing guided by specific indicator conditions, as is the case for HIV, was expanded to also include combined testing for HCV where appropriate. Combined HIV and HCV testing was found to be particularly feasible with high positivity rates among people presenting with STIs and those attending drug and alcohol management centres. The results of these interventions are further described in article 2 [9] and 3 [10]. At the policy level, this was supported by the development of the first integrated testing guidance for HIV and viral hepatitis, published by ECDC in December 2018 [11].

Since March 2020, many testing services have been affected by the COVID-19 pandemic. As a consequence, the Spring ETW in May 2020, focused on virtual activities and on the adaptations of testing services. So far, evidence shows a decline in the number of tests for infectious diseases other than for SARS-CoV-2, both in health care and community facilities [12, 13], which will be extremely important to monitor in future.

The acceptability and usability of self-tests for HIV was investigated in Lithuania and Italy respectively, and presented in article 4 [14], showing that 75% of online survey respondents in Lithuania stated they would likely buy and use an HIV self-test in the future, citing confidentiality, privacy and a rapid result as the main reasons, while the most commonly cited barrier was price. In Italy, 71% of first time HIV self-testers were satisfied with their test experience and said they would use an HIV self-test in the future: the most commonly cited reason being a rapid result. As a diagnostic tool, self-testing has the potential to reach people who might not attend established services and it is a recommended element in a testing strategy for key populations that should undertake regular testing [15, 16]. However, self-tests for HIV are regulated differently across Europe and less than half of countries in Europe (47%, 15/32) have fully implemented HIV self-testing and the price (Euros 20–25) remains a major barrier to access. Also, according to UNAIDS Global AIDS Monitoring reporting from 10 countries in Europe, only around 40.000 kits have been distributed by the public sector in 2019 [17]. Further, self-tests for other infections are only now starting to be considered, which is likely to represent a missed opportunity for timely diagnosis and important individual and public health benefits of early treatment. A number of usability, feasibility and acceptability studies are underway on hepatitis C self-testing, led by the Foundation for Innovative New Diagnostics [18] and World Health Organisation with the aim to develop guidance for implementation in early 2021, and with great potential for further integration of testing for multiple diseases in the future.

## Combination prevention

Article 5 [19] and 6 [20] present results from the INTEGRATE workstream focused on partner notification and the development of a web and mobile application designed to enhance the effectiveness of combination prevention by integrating HIV, hepatitis, STIs and TB in a single user-friendly information and communications technology (ICT) tool. For this purpose, a desk review was conducted to map existing online tools, revealing that to date most of them had been launched as disease specific aids. In response, INTEGRATE developed the application – “RiskRadar for HIV, hepatitis, STIs and TB” – the first of its kind to provide integrated information, risk assessment, test finders and an anonymous partner notification service for all four disease areas. RiskRadar succeeded in integrating prevention information and messages for multiple diseases in one user-friendly tool available for both android and iPhones, supporting ongoing efforts to address missed opportunities for multiple disease messaging and testing. It is presently available in four EU languages (Croatian, English, Italian and Lithuanian).

Article 6 [19] outlines the barriers and results on integrating partner notification and contact tracing services across Europe. The challenge of partner notification and contract tracing is that they are under the governance of different agencies in different countries, and indeed different systems have been set up within countries, depending on the local health authority. While there is clear national guidance on contact tracing for tuberculosis in most EU countries, the situation is different for the other infections – HIV, viral hepatitis and STIs. Partner notification should be part of best practice for all the conditions and ‘joined up’ pathways to care need to be developed between the different hospital, public health, and community agencies dealing with these diseases.

In article 7 [21], results from a patient experience survey undertaken in Romania and Spain among people living with HIV is presented. Results show that while health-related quality of life (HrQoL) was good overall in a sample of people accessing HIV care, people with HIV reported symptoms of anxiety/depression more frequently than the general population in both countries. Health concerns were highlighted as a key area of concern for people with HIV, despite high levels of anti-retro viral treatment coverage and adherence. Worse HrQoL was more likely to be reported by people with non-HIV related health conditions and financial instability. This study highlights the importance of monitoring HrQoLin people with HIV due to the chronic nature of the disease. In this highly-treatment experienced group, disparities were found, particularly highlighting mental health as an area which needs more attention in order to improve the well-being of people with HIV.

## Integration of data sources

Although community-based testing for HIV as well as for some other STIs and for hepatitis B and C has recently increased in most European countries, in some accounting for up 30% of reported new HIV cases, data from these services are seldom included in national monitoring and evaluation (M&E) information systems and disease specific surveillance reports [22]. INTEGRATE addressed gaps in surveillance of national testing in a workstream in collaboration with the European Centre for Disease Prevention and Control (ECDC), highlighted in article 8 [23] and 9 [24]. A minimum set of indicators for community testing has been proposed for inclusion in national surveillance and M&E systems through the Dublin Declaration monitoring system in order to provide a better overview of progress and to monitor effectiveness of different interventions [11, 22, 25]. Moreover, pilot studies aiming at increasing the integration of data derived from community testing into national surveillance and M&E information systems were implemented in Estonia, Latvia, Poland, Serbia, Slovenia and Spain. Their results and recommendations presented in this supplement and the experiences described will support such data integration in other European countries. ECDC included questions from the minimum list of community M&E indicators into their Dublin Declaration questionnaire 2020 to contribute to evidence-based community testing policies in European countries.

## Country responses - how to foster collaborations across diseases areas

Four national multi-stakeholder meetings were arranged in 2019 (Rome, Italy; Vilnius, Lithuania; Warsaw, Poland and Zagreb, Croatia) with the aim to foster cross-disciplinary and cross-disease-area collaborations at the national level. Results from these meetings are presented in article 10 [26] and show how multi-stakeholder discussions and in-depth analysis of strategies to address current gaps can foster synergies between stakeholders and a common national effort to seek solutions. Meeting outcomes raise awareness for how disease ‘silos’, often reflecting the mandate of organisations, are hampering synergies and implementation of integarted approaches.

On the ground, integration across diseases needs to consider the different levels of the health system – and the involvement of community-based programmes- across the continuum of care, but it is often hindered by practical challenges, local relationships, regulatory frameworks and legal barriers. Nevertheless, the INTEGRATE Joint Action has demonstrated that there is a high level of support among national stakeholders across Europe for improving the cross-disease integration of HIV, hepatitis, STI and TB services. In many instances, the participation in an EU co-funded Joint Action like INTEGRATE, bringing national experts together with agency representatives and international experts helped improve collaborations between services and disease areas, and resulted in better harmonization of services, data collection and data sharing. Cross-border collaborations such as INTEGRATE and the support from European Union agencies such as the ECDC and EMCDDA are key to enable the sharing of experiences, transfer lessons and support or facilitate a change from the current siloed approach to an integrated one.

The need for integration has been further heightened since the declaration of the COVID-19 pandemic in March 2020. As the impact of the pandemic on the testing for other infectious diseases becomes clearer, the need for collaboration and integration, for making different testing modalities (like self-testing and community-based testing) available as well as the need for establishing timely and sensible monitoring systems to understand evaluate and improve the effectiveness of HIV, viral hepatitis and STI strategies becomes increasingly evident and a challenge for European Health Services.

## Data Availability

Not applicable.

## Abbreviations

COVID-19: Corona Virus Induced Disease 2019
ECDC: European Centre for Disease Prevention and Control
EMCDDA: European Monitoring Centre for Drugs and Drug Addiction
ETW: European Testing Week
EU: European Union
Euro HIV EDAT: Operational knowledge to improve HIV early diagnosis and treatment among vulnerable groups in EuropeHCV - Hepatitis C virus
HIV: Human immunodeficiency virus
HIV-COBATEST: HIV community-based testing practices in Europe
HrQoL: Health-related quality of life
ICT: Information and communications technology
INTEGRATE: Integrating prevention, testing and linkage to care strategies across HIV, viral hepatitis, TB and STIs in Europe
M&E: Monitoring and Evaluation
NGOs: Non-governmental institutions
OptTEST: Optimising testing and linkage to care for HIV across Europe
SARS-CoV-2: Severe Acute Respiratory Syndrome Coronavirus 2
STI: Sexually transmitted infection
TB: Tuberculosis
UNAIDS: The Joint United Nations Programme on HIV/AIDS
WHO: World Health Organization
